# PhysiBoSS 2.0: a sustainable integration of stochastic Boolean and agent-based modelling frameworks

**DOI:** 10.1038/s41540-023-00314-4

**Published:** 2023-10-30

**Authors:** Miguel Ponce-de-Leon, Arnau Montagud, Vincent Noël, Annika Meert, Gerard Pradas, Emmanuel Barillot, Laurence Calzone, Alfonso Valencia

**Affiliations:** 1 Life Science, Barcelona Supercomputing Center (BSC), 1-3 Plaça Eusebi Güell, 08034 Barcelona, Spain; 2 Institut Curie, Université PSL, 26 rue d’Ulm, 75248 Paris, France; 3 INSERM U900, Paris, France; 4 Mines ParisTech, Université PSL, Paris, France; 5 ICREA, 23 Passeig Lluís Companys, 08010 Barcelona, Spain

**Keywords:** Software, Software, Multicellular systems, Computer modelling

## Abstract

In systems biology, mathematical models and simulations play a crucial role in understanding complex biological systems. Different modelling frameworks are employed depending on the nature and scales of the system under study. For instance, signalling and regulatory networks can be simulated using Boolean modelling, whereas multicellular systems can be studied using agent-based modelling. Herein, we present PhysiBoSS 2.0, a hybrid agent-based modelling framework that allows simulating signalling and regulatory networks within individual cell agents. PhysiBoSS 2.0 is a redesign and reimplementation of PhysiBoSS 1.0 and was conceived as an add-on that expands the PhysiCell functionalities by enabling the simulation of intracellular cell signalling using MaBoSS while keeping a decoupled, maintainable and model-agnostic design. PhysiBoSS 2.0 also expands the set of functionalities offered to the users, including custom models and cell specifications, mechanistic submodels of substrate internalisation and detailed control over simulation parameters. Together with PhysiBoSS 2.0, we introduce PCTK, a Python package developed for handling and processing simulation outputs, and generating summary plots and 3D renders. PhysiBoSS 2.0 allows studying the interplay between the microenvironment, the signalling pathways that control cellular processes and population dynamics, suitable for modelling cancer. We show different approaches for integrating Boolean networks into multi-scale simulations using strategies to study the drug effects and synergies in models of cancer cell lines and validate them using experimental data. PhysiBoSS 2.0 is open-source and publicly available on GitHub with several repositories of accompanying interoperable tools.

## Introduction

In systems biology, mathematical models and simulations play a crucial role in understanding complex biological systems^1^. Mathematical models provide a theoretical framework for studying the properties and behaviour of biological systems at different scales, including signalling and regulatory networks^2,3^ metabolism^4,5^ as well as models integrating different intracellular processes^6^. Beyond the cellular level of description, models and simulations are also used to study population dynamics in a given environment; some examples include simulating bacterial communities^7^, tissues^8^ and tumour growth^9,10^.

Different modelling frameworks are employed depending on the nature and scales of the system under study. For instance, signalling and regulatory networks can be simulated using Boolean modelling, an approach that employs logical rules and discrete time to describe how signals propagate through the molecular pathways and induce changes in the cell’s phenotype^2^. In the context of cancer research, Boolean modelling has been extensively used to uncover clues in a cell’s signalling network that lead to disease^3,11–13^. Moreover, Boolean models can be used to personalise treatment plans based on a patient’s specific genetic and molecular profile, improving the efficacy and outcome of the treatment^14,15^.

**Fig. 1:**
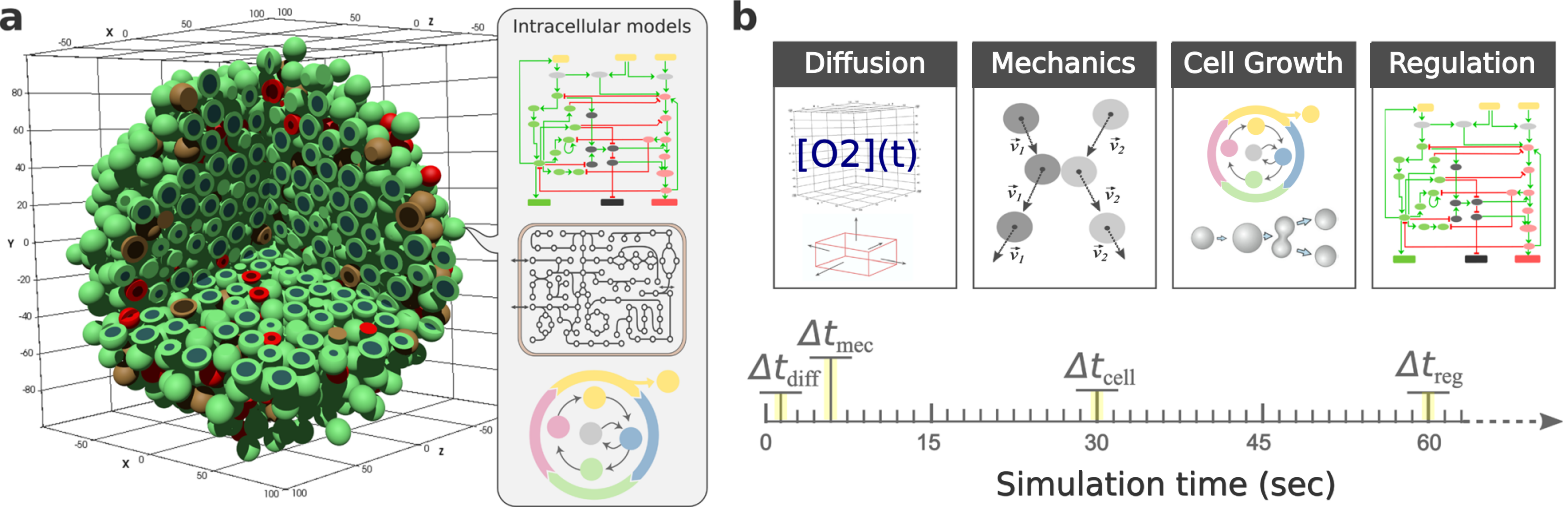
Multi-scale simulations in systems biology. **a** shows a schematic representation of an agent-based model of a 3D multicellular system in a microenvironment defined by the domain divided into fixed volumes together with examples of different intracellular models including signalling network, metabolism and cell cycle. **b** depicts examples of the different simulators and time scales for a multi-scale model including, the Δ*t*_*diff*_ time scale where diffusion, uptake and secretion processes are updated; Δ*t*_*mec*_ where the mechanics (movement and physical interactions) are updated; Δ*t*_*cell*_ in which cell processes such as volume, cell cycle and death models are updated; and Δ*t*_*reg*_ the regulatory time scale in which Boolean models are updated.

On the other hand, simulating the dynamics of a population of cells in a defined environment requires the use of a more general modelling framework, such as agent-based modelling (ABM)^10^. In these models, agents represent individual cells that can interact with each other and their environment (Fig. 1a). ABMs find extensive application in different domains such as microbial ecology^7,16^ and cancer research^17–19^.

In general, ABM frameworks use different types of solvers for simulating processes occurring at different scales, such as diffusion, mechanical interactions and cellular processes, e.g. growth and division, and thus are commonly referred to as multi-scale models (Fig. 1b)^9,10,20^. A good example is PhysiCell, a multicellular simulation framework composed of different solvers, such as a diffusion and transport solver used to simulate the chemical microenvironment and its interaction with the cells, a mechanical solver to simulate mechanical interaction between cells, as well as other components to simulate cellular processes, including growth, division, cell differentiation and death^21,22^.

Interestingly, PhysiCell has been extended to allow simulating cell signalling and regulatory pathways, allowing the study of the interplay between cell regulatory processes, the environment and population-level dynamics^23^. This extension named PhysiBoSS^23^ was in its version 1.0 a standalone multi-scale simulation software that was implemented by coupling an early version of PhysiCell together with MaBoSS^24,25^, a continuous-time Markovian simulator for Boolean models that describe the cell’s intracellular signalling and regulatory networks.

PhysiBoSS allows bridging microenvironment signals, such as drugs, their effect on signalling pathways and the resulting population-level phenotypes^23,26–28^. The introduction of this hybrid simulation framework was an important step toward the mechanistic multi-scale description of complex biological systems such as healthy tissues and tumours^9^. Nevertheless, in its original version, PhysiBoSS presented some design problems as it lacked a clear interface between these two tools which resulted in a software package that is hard to modify and extend without changing core functionalities, hindering its long-term maintenance and usability. Furthermore, it was designed and tested using a specific signalling model with a set of predefined phenotypes making it hard to adapt to different models and use cases.

**Fig. 2:**
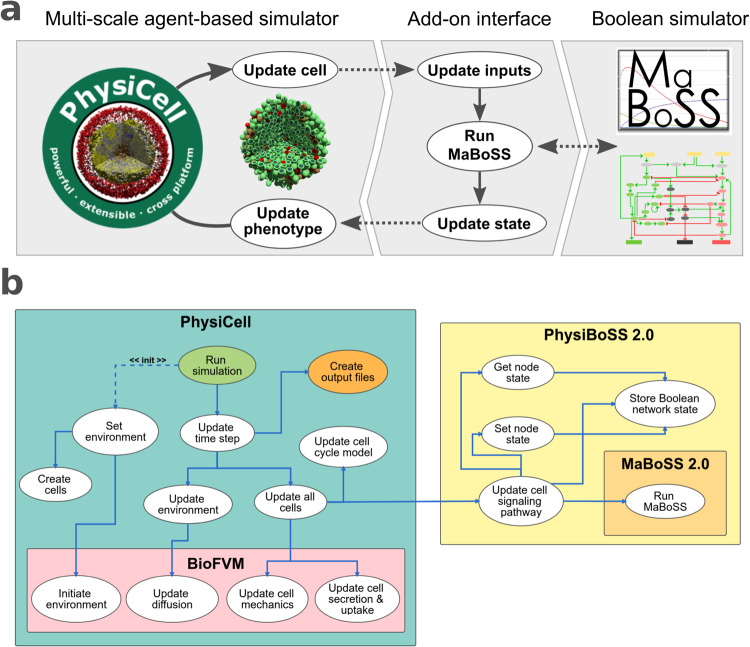
PhysiBoSS 2.0 add-on-based design. **a** shows a diagram of the add-on-based design of PhysiBoSS 2.0 that decouples PhysiCell and MaBoSS providing Boolean simulation functionality to individual cell agents in a maintainable manner. **b** depicts a high-level view of the PhysiCell and PhysiBoSS 2.0 and the communication between the different components.

Herein, we present PhysiBoSS 2.0, a new design and reimplementation of PhysiBoSS that solves the problems of its early version, extends its functionalities and enables a more flexible definition of models. PhysiBoSS 2.0 was implemented as an add-on component of PhysiCell that provides access to the MaBoSS simulator in a clear and transparent way. In this new design, both PhysiCell and MaBoSS are decoupled and therefore, can be upgraded independently (Fig. 2). Furthermore, the code was designed and implemented following the best practices guidelines for bioinformatics software development, ensuring its long-term maintenance and prolonging its lifespan^29^.

PhysiBoSS 2.0 includes new functionalities like allowing the use of highly customisable settings such as user-defined Boolean models and cell types defined in the configuration XML. We also present PhysiCell Tool Kit (PCTK) a Python package developed for handling and processing simulation outputs, generating summary plots and 3D renders based on POV-Ray^30^. In this work, we show different approaches for integrating Boolean models into multi-scale simulations using the examples presented in the original publication. Moreover, we have used PhysiBoSS 2.0 new features to study the drug effects and synergies in multi-scale simulations^26,27^ of a prostate cancer cell line, LNCaP, with six available drugs^31^. We have validated the single-drug treatments using experimental data and inspected closely the effect of drug dosages in the generation of heterogeneities in the cell population. Altogether, we show that PhysiBoSS 2.0 is a step towards the development of a PhysiCell add-on ecosystem of different types of models^32^ that allows for the scaling-up of simulations in exascale high-performance computing clusters^20,26,33^.

## Results

In this section, we first introduce the redesign of PhysiBoSS together with the newly implemented features and functionalities. Secondly, we present novel results as examples of the kind of experiments possible with PhysiBoSS 2.0. We showcase here two examples: the integration of different cell receptor models for coupling the presence of environmental signalling substrates to the intracellular Boolean model as well as the integration of pharmacodynamics and Boolean models to conduct *in-silico* drug screen studies.

### The new design of PhysiBoSS 2.0 allows for extended functionalities

PhysiBoSS is a multi-scale multicellular simulation framework that integrates PhysiCell^21^ and MaBoSS^24,25^, enabling the simulation of cell signalling and gene regulatory networks in each cell agent. In this way, cell agents can integrate environmental and genetic signals and respond according to their internal Boolean model dynamics (see Fig. 2a). Therefore, PhysiBoSS bridges the molecular level description of cell signalling and gene regulation with the surrounding microenvironment and the population dynamics, allowing the coupling between these different scales. For instance, PhysiBoSS enables simulating drug studies such as adaptive therapy in cell populations while considering the role of heterogeneity among the cells and the environment and their role in the emergence of drug resistance^27^.

In its original version, PhysiBoSS 1.0^23^ was implemented merging MaBoSS and PhysiCell by adapting several core classes of the latter, resulting in a new standalone simulation framework. This design proved to be very difficult to maintain and to keep it aligned with the latest version of the original software components. For the same reason, bug fixing and the development of new features was also a difficult task. To solve these issues, PhysiBoSS 2.0^34^ was re-designed and re-implemented from scratch as an add-on interface which decouples PhysiCell and MaBoSS minimising dependencies and fixing many design problems of version 1.0 (Fig. 2b and Supplementary Fig. 1). The new add-on-based design was conceived with the PhysiCell’s developer team and aims to simplify the tool’s maintenance and enable the independent upgrade of the different software components, something not possible in PhysiBoSS 1.0 (see Supplementary Fig. 2). Therefore, with PhysiBoSS 2.0 new implementation we aim to overcome several design problems from its predecessor version 1.0.

PhysiBoSS 2.0 uses the latest versions of MaBoSS and PhysiCell and thus incorporates all the new features and functionalities provided by the individual frameworks. For instance, from version 1.9 PhysiCell allows to keep track of substrates internalised within cells through a transport process. In PhysiBoSS 2.0, these quantities can be used as signals or inputs of a Boolean model, e.g. a drug that inhibits a target protein. Furthermore, it is also possible to implement rule-based cell–cell and cell–extracellular matrix contact behaviours that trigger signals that propagate through a Boolean model^28^. On the other hand, the latest MaBoSS release (2.4.0) allows working with Boolean models of unlimited size as well as having these models defined in the SBML-qual standard.

Among other new features, it is now possible to set up simulations in the XML configuration file (see options in Table 1) which include different cell types that are associated with specific Boolean models. Moreover, we have standardised the integration of the Boolean model by using custom modules that connect intracellular variables to agent-based ones (discussed in Section *PhysiBoSS 2.0 reproduces original results and allows to test new transport mechanisms*). Table 1 summarises all the new features that can be defined by the user using the extended PhysiCell XML configuration file.

**Table 1:** PhysiBoSS intracellular model configuration.

XML tag	Description	Default
bnd_filename	Path to the MaBoSS BND file	None
cfg_filename	Path to the MaBoSS CFG file	None
time_step	Time step for the update of the intracellular model	12
scaling	Parameter to adapt the time-scale of the MaBoSS model	1
time_stochasticity	Parameter to configure stochastic update time	0
initial_values	List of initial_values, to modify the initial value of a MaBoSS model node	Empty
mutants	List of mutants, to modify the value of a MaBoSS model node during the whole simulation	Empty
parameters	List of parameters, to modify the value of a MaBoSS model parameter	Empty

Finally, different sample projects and templates are provided with the source code to facilitate the implementation of new models (see Supplementary Section 1.4). PhysiBoSS 2.0 is written in C++ and the implementation is described in further detail in the Supplementary Methods, together with the introduction of a template project for new users. PhysiBoSS 2.0 code is open-source and distributed under BSD 3-clause license. The links to the repositories of the different versions are provided in Table 3. In addition, we also provide a nanoHUB GUI-based tool implementing an example model to ease its use at https://nanohub.org/tools/pba4tnf/.

### Handling and processing simulation outputs

Processing, analysing and visualising multi-scale simulations’ output is a non-trivial task as it requires handling and integrating several output file formats into aggregated easy-to-use data frames as well as using 3D rendering libraries to generate visual representations of the system. For this reason, in addition to PhysiBoSS 2.0, we have developed the PhysiCell Toolkit (PCTK) a Python-based package that includes a library and command-line scripts to process and analyse simulation outputs. Although there are already available tools for handling PhysiCell outputs (such as https://github.com/PhysiCell-Tools/python-loader), with PCTK we aim to gather and organise different pieces of Python code that have been recurrently used in different projects involving PhysiCell and PhysiBoSS. The package implements different functionalities to parse and handle the MultiCellDS file format^35^ and uses an efficient schema to process the output files containing the cells and microenvironment data. Moreover, on top of this module, we have implemented a command-line tool to ease the processing of simulations’ output and create basic plain text (CSV) data-frames as well as to generate basic plots, including time courses of the number of alive and dead cells. Additionally, it also provides command-line functionalities to allow users to generate POV files used as inputs for the 3D rendering of the multicellular models using POV-Ray^30^. Therefore, PCTK can be used both as a callable library and as a stand-alone command-line tool; the code is open-source and distributed under BSD 3-clause license and the link to the repository can be found in Table 3. Documentation and examples are also provided in the PCTK repository^36^.

### PhysiBoSS 2.0 reproduces original results and allows to test new transport mechanisms

The new functionalities of PhysiBoSS 2.0 allow for better modularity and ease of reuse of functions. These allow, for instance, researchers to focus on the important step of defining the interactions between variables of the intracellular Boolean models and the ones from the cell agent and environment. Depending on the level of detail desired, different approaches can be used, but usually, the problem can be split into two different sub-problems: how to couple cell and environmental signals (continuous variables) into the Boolean input nodes and how to use the value of the Boolean output nodes to control the behaviour of the cell agents. In general, the mapping of continuous variables into a Boolean value requires the use of a transfer function. The simplest case is the use of a step function *H*(*x*) which returns 1 when *x* ≥ *θ* and 0 otherwise, for a given threshold *θ*, as we had in ref. ^23^. On the other hand, using the value of the Boolean model’s output node to control the cell agent behaviour can be done by modulating the specific rates of the cell cycle or death models, as well as by triggering custom rules defined by the modeller (see ref. ^28^).

To test and validate the new PhysiBoSS 2.0 and to show how the different submodels can be integrated, we have re-implemented the different models presented together with the original PhysiBoSS 1.0 and replicated all the results reported by ref. ^23^. In Letort et al., we implemented a multi-scale model of 3T3 fibroblast spheroids to investigate the complex dynamics observed when tumour cells are exposed to different regimes of tumour necrosis factor (TNF)^37^. The model integrates the Cell Fate Boolean network^3^ inside the cell agents to simulate the growth of a spheroid of cancer cells under different treatment regimes, which correspond to the supply of TNF pulses with different frequencies, duration and concentrations.

**Fig. 3:**
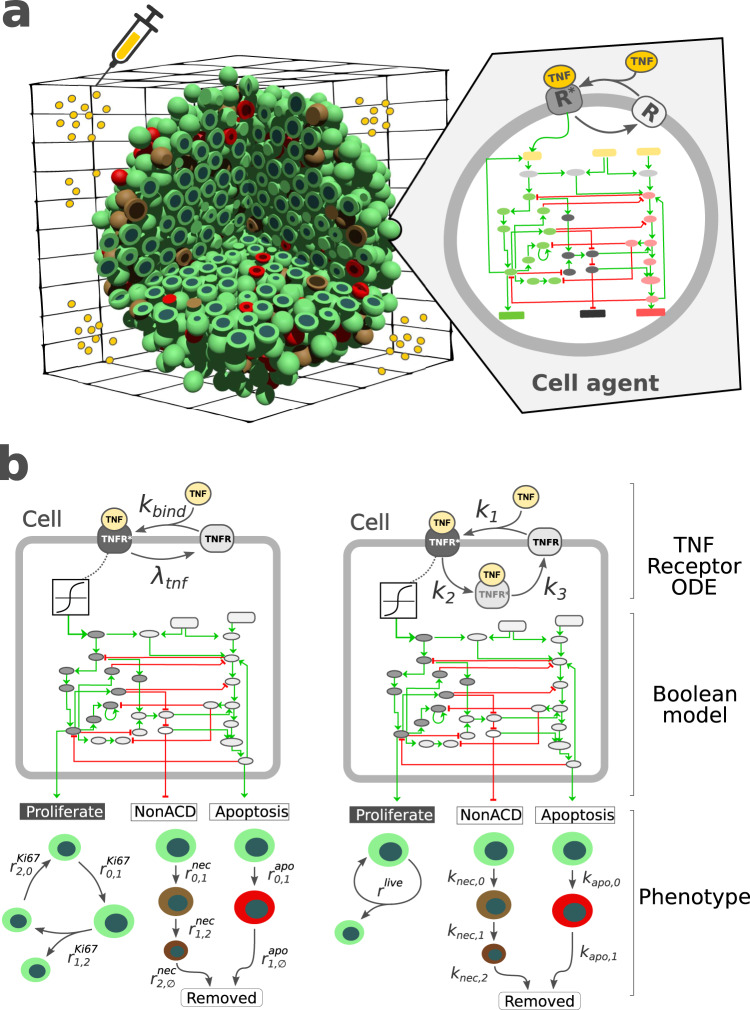
Different implementations of the multi-scale TNF models. **a** schematically represents the multi-scale model used to explore the 3T3 fibroblast spheroids growth dynamics under different TNF exposure regimes. **b** shows the intracellular models used in the PhysiBoSS original publication (left) and the extended version implemented in PhysiBoSS 2.0 (right).

**Fig. 4:**
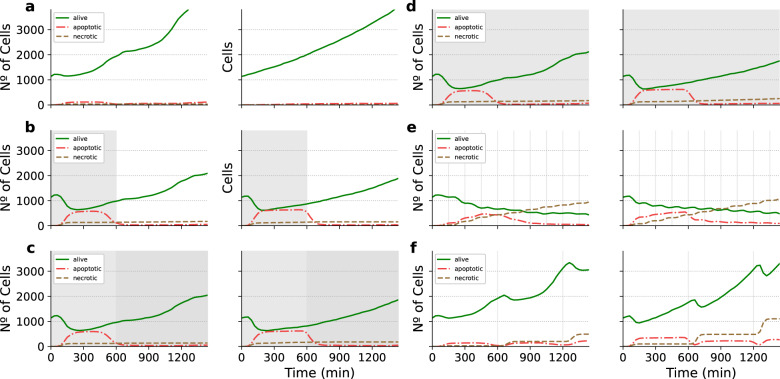
Pair comparison between results obtained using PhysiBoSS 1.0 and PhysiBoSS 2.0. The plots represent population growth curves for the same TNF pulse in silico experiments reported in the PhysiBoSS 1.0 (left column) and 2.0 (right column). Each panel corresponds to a different in silico experiment. **a** No TNF added; **b** single pulse of 0.5 ng/mL for 10 h (600 min); **c** single pulse of 0.5 mg/mL for 10 h followed by a second pulse of 5 mg/mL for 14 h; **d** continuous pulse of 0.5 ng/mL throughout the 24 h the experiment last (1440 min); **e** TNF pulses of 0.5 ng/mL and duration of 10 min at intervals of 150 min; and **f** TNF pulses of 0.5 ng/mL and duration of 10 min at intervals of 600 min. Vertical grey patches represent the TNF pulses.

We re-implemented the model in PhysiBoSS 2.0 using an extended version which includes a more detailed description of the TNF-receptor binding mechanisms^27^. Figure 3 shows a schematic representation of the multi-scale model as well the difference between the TNF receptors and growth model used in refs. ^23,27^. Details of the model formulation and implementation can be found in the Supplementary Methods. The experiments consist of an initial tumour spheroid of ~1000 cells that are exposed to different concentrations and regimes of TNF. We run all the different experiments using the same simulation setups to replicate Fig. 4 from ref. ^23^ using both the re-implemented model and PhysiBoSS 2.0 and the original code (additionally, we have also the matched results of the same model in the original PhysiBoSS code and in PhysiBoSS 2.0 in Supplementary Section 2.1). The comparative results are presented in Fig. 4, where each panel shows the output generated by each of the two PhysiBoSS versions.

The results show that the simulations obtained with PhysiBoSS 2.0 qualitatively reproduce the results reported by Letort et al. (2019). Specifically, the new implementation correctly reproduces the pattern observed when cells are exposed to a continuous supply of TNF causing cells to become resistant to the effect of the cytokines (Fig. 4b–d). Moreover, the model implemented in PhysiBoSS 2.0 also correctly predicts the reduction of the tumour when cells are exposed to short pulses of TNF at a frequency of 150 min (Fig. 4e) as well as the ineffective regime of short pulses of TNF at a frequency of 600 min (Fig. 4f). Furthermore, we can observe that PhysiBoSS 2.0 better captures the exponential cell growth that was somehow biphasic in the original PhysiBoSS (Fig. 4a). The differences observed in all the experiments are mainly due to the differences in how the TNF binding and the cell cycle models were implemented in the new version (see Fig. 3b). Nevertheless, implementing the same cell cycle and transport models retrieved the same results (Supplementary Section 2.1). Beyond the difference in the transport mechanism, the new version was implemented with a clear separation of the different submodels as well as the interfaces to connect them and it can be used as a sample project to implement new models since the code is packed with PhysiBoSS 2.0.

### Simulating drug screening studies and drug synergies

**Fig. 5:**
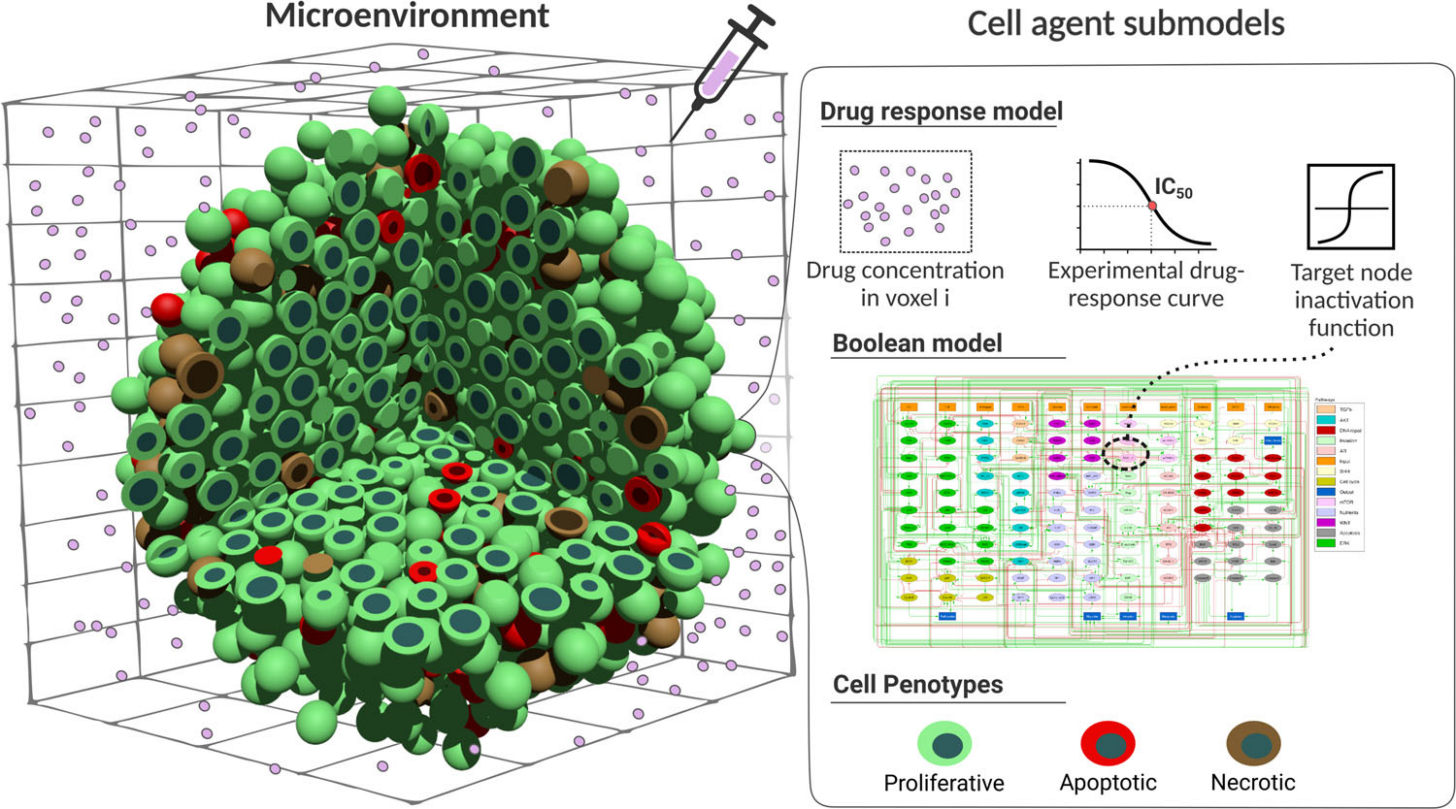
Multi-scale simulation of LNCaP prostate cancer cell line and combinations of drugs. Overview of PhysiBoSS 2.0 simulation framework. Drugs in the microenvironment affect the cells' behaviours according to an experimental drug-response curve. Depending on how a specific drug affects a specific cell line, the node targeted by the drug is inhibited at a given rate affecting the cell’s phenotype probabilities, allowing for a tailored simulation of drugs and cell lines.

We used PhysiBoSS 2.0 new functionalities to portray the phenotypic characteristics of cell lines and drugs’ effects and synergies by using personalised Boolean models^14,31^ and GDSC dose-response profiling data^38^. The models described in the previous sections rely on a dynamic model that mechanistically describes the interaction between the signal molecule (TNF) and its target (TNF-receptor) and a step function to transfer the level of TNF bound in the membrane to the corresponding Boolean input node. However, the detailed mechanism and kinetics of how drugs interact with their targets are largely unknown. For such cases, in PhysiBoSS 2.0 users can leverage experimental dose-response curves, such as the ones from GDSC, to include the effect of drug concentrations when inhibiting a node of the intracellular signalling model (Fig. 5).

Therefore, when a drug is available at a specific concentration in the surroundings of a cell, the *cellviability* (alive cells after drug treatment) is looked up in the dose-response curve that is specific to the cell line and the drug, as described in ref. ^38^ using fluorescent intensity with drug-treated and untreated samples. Then, the corresponding drug target node in the Boolean model is inhibited with a probability of 1 − *cellviability* (see “Methods” for further details). This ensures that for a concentration of IC90, for example, a node inhibition would be obtained in 90% of the cases and that an IC10 would lead to a node inhibition with 10% probability (see an example dose–response curve in n Supplementary Fig. 4. Here, we showcase examples that integrate pharmacodynamics and Boolean models to study single drug effects, drug combinations and their synergies.

#### Single drug screening studies

Using PhysiBoSS 2.0, we simulated spheroids of ~1000 cells of the prostate cell line LNCaP for seven days with six drugs (Table 2) in a three-dimensional domain where the drugs were supplied from the domain boundaries (the box walls) and diffused to the centre of the simulation space e (Supplementary Fig. 15). We tested five different concentrations for each drug corresponding to the IC10, IC30, IC50, IC70 and IC90. This rather simple setup leads to varying drug availability and effects throughout the different layers of the tumour and, therefore, heterogeneously affects the cancer cells depending on their location and if they are surrounded by cells or in contact with the microenvironment (see the following sections).

**Table 2:** Drug–target pairs used to perform the drug simulations on the LNCaP-specific Boolean model.

Gene targets	Node	Drug name	Drug GDSC ID
AKT1, AKT2, AKT3	AKT	Ipatasertib	1924
EGFR	EGFR	Afatinib	1032
MAPK1	ERK	Ulixertinib	2017/1908
HSP90AA1, HSP90AB1, HSP90B1, HSPA1A, HSPA1B, HSPB1	HSPs	Luminespib	1559
MAP2K1, MAP2K2	MEK1_2	Selumetinib	1736
PIK3CA, PIK3CB, PIK3CG, PIK3CD, PIK3R1, PIK3R2, PIK3R3, PIK3R4, PIK3R5, PIK3R6, PIK3C2A	PI3K	Pictilisib	1058

**Fig. 6:**
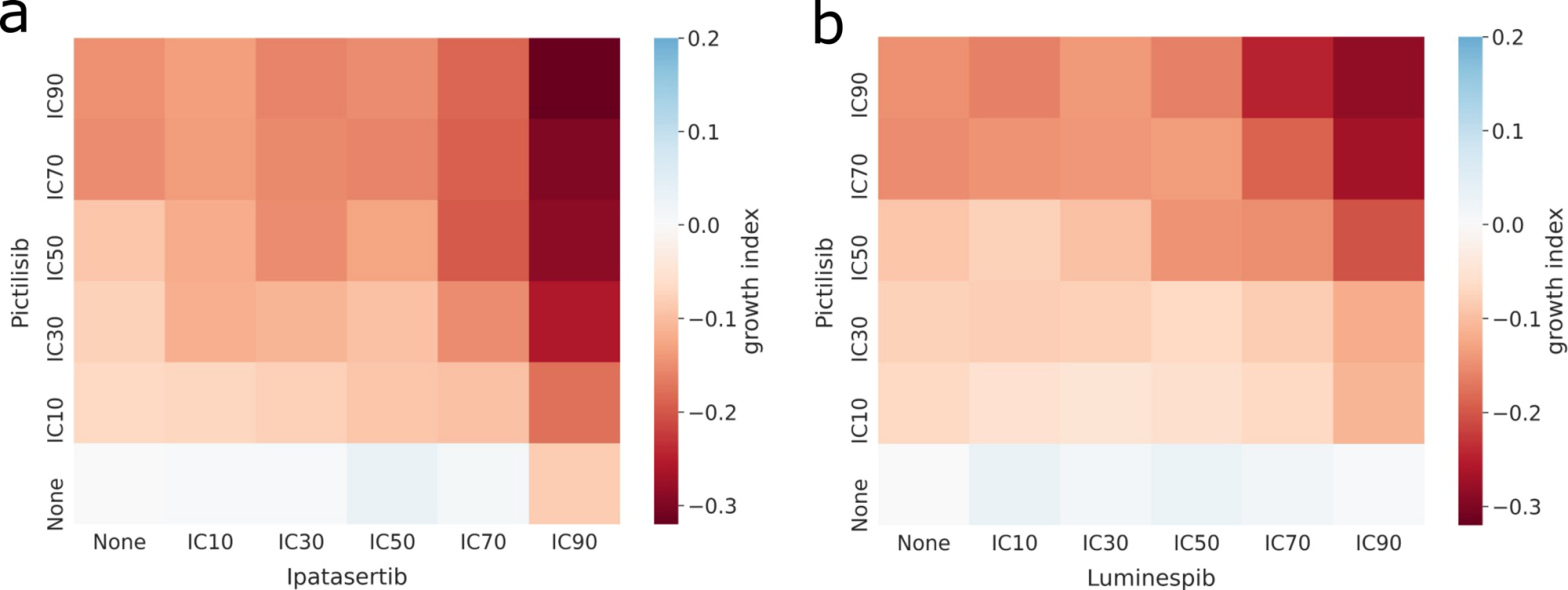
Growth index of the multi-scale simulations with different drug combinations with respect to the untreated LNCaP. **a** Pictilisib and Ipatasertib drug combination; **b** Pictilisib and Luminespib drug combination. Each simulation was replicated 10 times. For each combination, the growth index was obtained by taking the log2 of the ratio between the median AUC upon drug administration and the median AUC of the untreated simulations. “None” row and column means the cells were not treated with the drug. White colour means no growth behaviour change upon drug administration, blue means the drug increased the growth and red means that the drug diminished the growth of the cells. For a complete figure of all the combinations, refer to Supplementary Fig. 9.

We simulated five concentration simulations for all six drugs (Supplementary Fig. 9) and analysed the significance of growth behaviour changes for each drug simulation, finding two out of six drugs significant: Ipatasertib (targeting AKT) and Pictilisib (targeting PI3K) with *P* ≤ 0.0001 (Kruskal–Wallis rank sum test, Fig. 6a and Supplementary Table 3). For instance, when simulating Pictilisib dosages, we can see that starting with IC10, the Growth Index decays until it is minimised at IC90 (Fig. 6a).

#### Double drugs’ screening studies

**Fig. 7:**
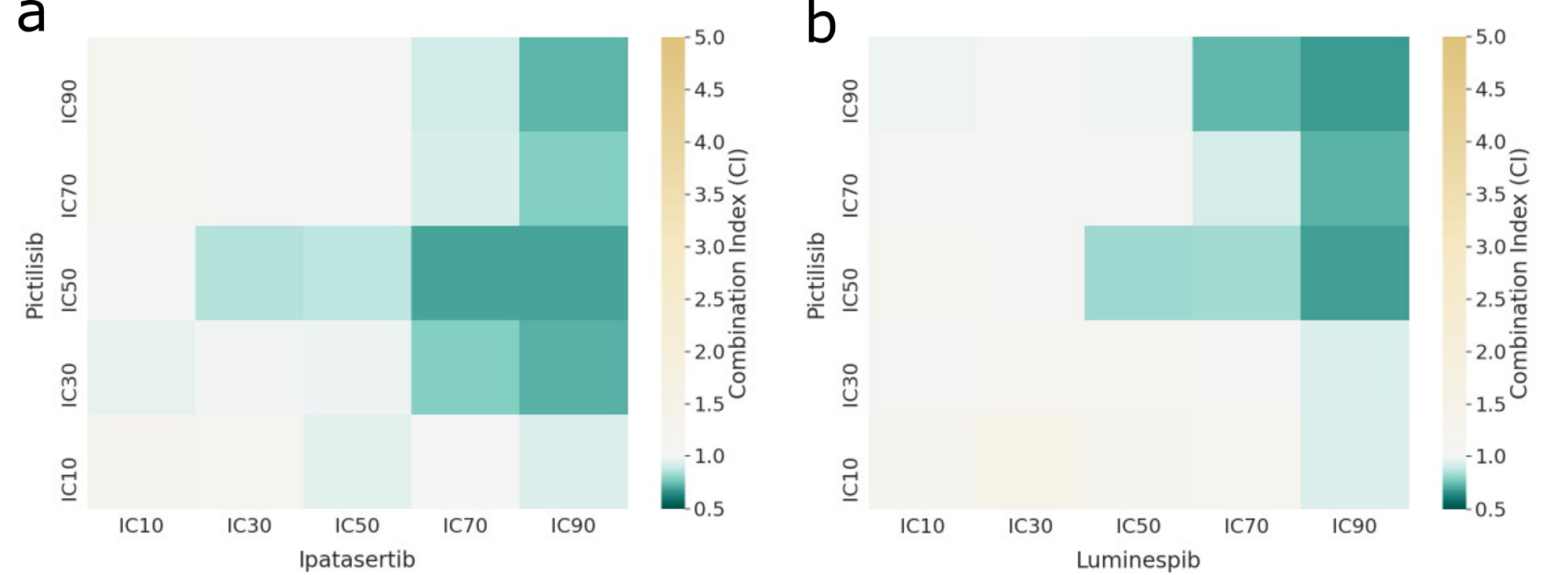
Bliss independence Combination Index (CI) of the multi-scale simulations of LNCaP with different drug combinations. **a** Pictilisib and Ipatasertib drug combination; **b** Pictilisib and Luminespib drug combination. White colour indicates an additive effect, green colour a synergistic effect and yellow an antagonistic effect. For a complete figure of all the combinations, refer to Supplementary Fig. 11.

Furthermore, PhysiBoSS 2.0 also enables combining the administration of more than one drug allowing the simulation of drug-synergy studies. As with the single-cell studies, we used PhysiBoSS 2.0 to simulate spheroids of ~1000 LNCaP cells with combinations of the aforementioned six drugs with their five different concentrations (Table 2). We identified two combinations of drugs as interesting: Pictilisib with Ipatasertib and Pictilisib with Luminespib (targeting HSPs) with a significant *P* value ≤0.0001 in the Kruskal–Wallis rank sum test. For both drug combinations, we can observe a gradual growth inhibition with the drug concentration (Fig. 6) and the indication of drug synergy as combined inhibition levels outdo single effects (Fig. 7). While the drug combination Pictilisib and Luminespib requires IC70 and IC90 for both drugs to obtain a Growth Index of around −0.3, the drug combination Pictilisib and Ipatasertib reaches similar values already with much lower concentrations for Pictilisib, IC30 or IC50 (Fig. 6a, b and Supplementary Fig. 9).

In addition, we studied the Bliss independence of these combinations (Fig. 7 and Supplementary Fig. 11) and found complex synergies in both cases. Synergy values reach their maximum for Pictilisib and Luminespib with high drug concentration values of both compounds being Luminespib the drug driving the synergy. For Pictilisib and Ipatasertib, on the other hand, synergy values peak at IC50 and IC70 for both compounds. Comparably, high drug concentrations of Ipatasertib especially drive the synergy values. For both drug combinations synergy values strongly decrease or even turn into slight antagonism with low drug concentrations. Such varying combinatorial effects depending on the drug concentration have previously been observed experimentally. For instance, drug combinations can be both antagonistic and synergistic at the same time which can be described with the help of exposure-response surfaces^39^. These surfaces can help in choosing drug concentrations for combinatorial therapies. The complete study of LNCaP and six drugs (Ipatasertib targeting AKT, Luminespib targeting HSPs, Pictilisib targeting PI3K, Afatinib targeting EGFR, Ulixertinib targeting MAPK1, Selumetinib targeting MAP2K1 and MAP2K2) can be found in Supplementary Section 2.2.3.

#### Studying the heterogeneity of drug screening simulations

The integration of drug simulations in PhysiBoSS allows for the expansion of the study of the heterogeneous behaviours emerging from the cells. In Letort et al.^23^, we studied the effect of different genetic backgrounds, such as mutants cFLIP+ IKK+ and CASP3+ Cytc+, in their response to TNF treatments, as we have replicated in PhysiBoSS 2.0 (Supplementary Sections 2.1 and 2.2 and Supplementary Fig. 6). In addition to the genetic perturbations, PhysiBoSS 2.0 can be used to study the heterogeneity in the cells’ response to drug inhibition in a population of cells with a common genetic background. In this scenario, heterogeneity comes from a non-homogeneous microenvironment and is caused by the drug penetration into the cell population resulting in varying substrate availability in different regions of the spheroid of cells that affect the drug’s effectiveness. In our simulations, the drug is supplied on the simulation boundaries assuring a uniform administration of the drug. As cells act as sinks for the drug, by uptaking it at a given rate, cells in the outskirts of the spheroid will have more available drugs than the ones in the spheroid centre (see Supplementary Fig. 15).

**Fig. 8:**
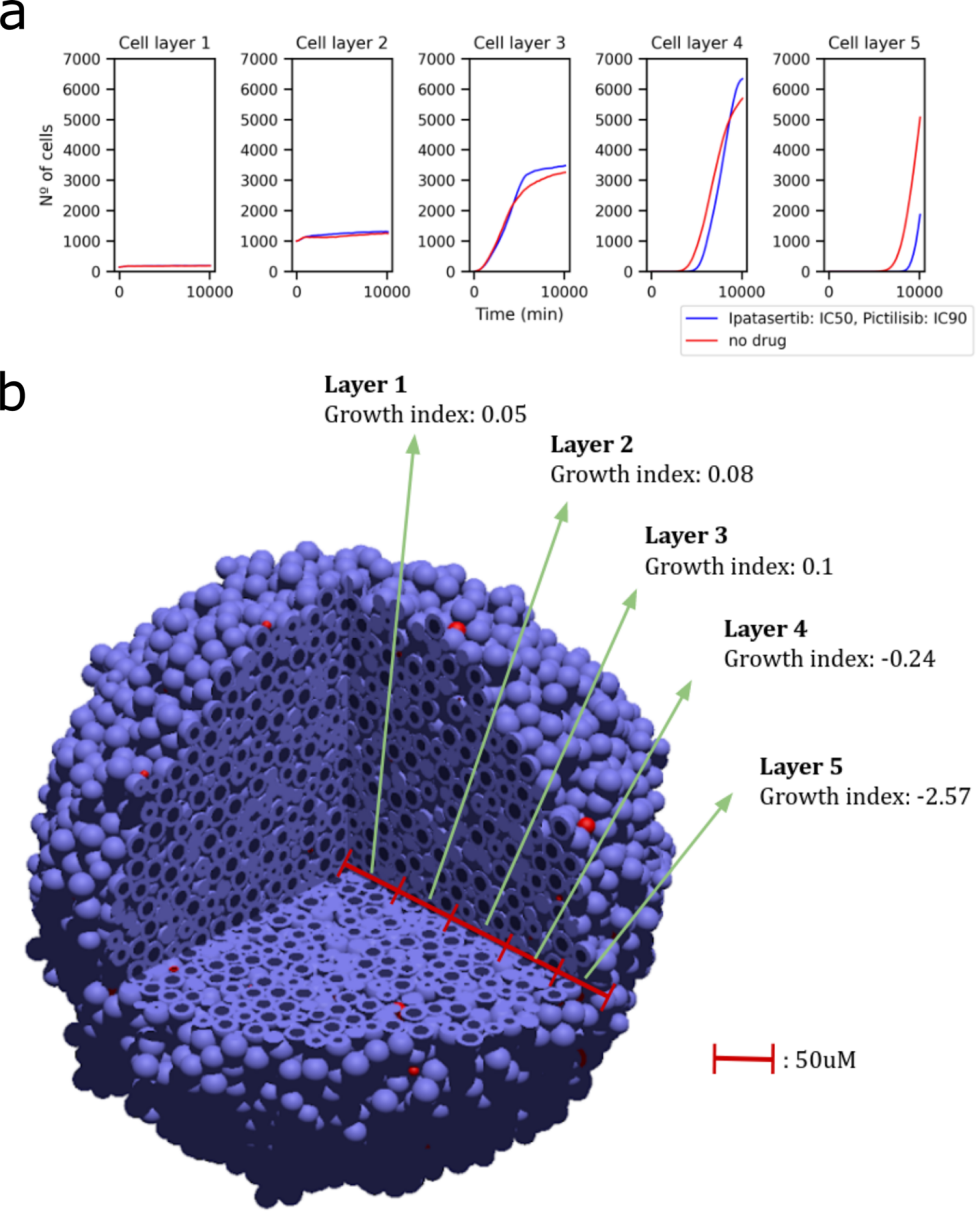
Heterogeneity of drug screening simulations. **a** shows the growth curves for the no-drug simulation and the drug simulation with Ipatasertib (IC50) and Pictilisib (IC90) separated into sphere layers. By taking the distance to the tumour centre, we define five 50 μm-thick spherical layers: layer one is the innermost layer and layer five corresponds to a distance from 200 to 250 μm from the tumour centre. From these growth curves, the AUC values and growth indices are calculated for each layer separately. **b** shows a 3D representation of the cell population growth indices of the five 50 μm-thick spherical layers at the end of the simulation. Layer 1 is the innermost layer from the centre of the tumour to a distance of 50 μm from the centre. Blue cells are living cells and Red cells are apoptotic.

To study the effect of the position of the cell on their response to the drugs, we simulated a spheroid of a radius of 100 μm with drug administration on the boundaries with 10 replicates. We studied the growth rate of the different layers of the spheroid by splitting the spheroid into 50 μm-thick layers from their Euclidean distance to the tumour centre. At the beginning of the simulations and as the initial radius is 100 μm, the cells are only in layers 1 and 2. Then the cells start growing into layer 3 at around 200 min in the simulation, then layer 4 at around 5000 min and then 5 at around 8000 min. Figure 8a depicts the number of cells for each spheroid layer of one simulation with and without drugs. Replicating this experiment yielded similar results.

The growth curves obtained when applying Ipatasertib (IC50) and Pictilisib (IC90) (Fig. 8a, blue) and without drug (Fig. 8a, red) are almost identical for the inner layers 1 and 2, indicating the absence of drugs in the central tumour regions. Layer 4, which starts at 150 μm from the centre, is reached before by the cells with no drug (red curve in Fig. 8a) than by the cells treated with drugs (blue curve). This difference increases in layer 5, which starts at 200*μ*m from the centre, where the no-drug red curve starts much sooner than the drug blue curve. In these layers, the cells are closer to the drug source and more available drug that is killing them by apoptosis.

Interestingly, the reduced survival of layers 4 and 5 of the drug simulations is causing a slight increase in growth rate in layers 1–3 when compared to the no-drug simulation. We think this increased growth in the inner layers can be explained by the increase in available space. As drugs reduce the number of cells in outer tumour regions, it leave more space for cells in inner tumour regions to have space to proliferate. We can observe in layers 3 and 4 the intercept point of when the tumour reaches a size when not enough drug can reach the cells and the reduced amount of surrounding cells increases the available space to divide. Finally, the no-drug simulations have scarce space in the inner layers, causing the cells to have an outwards pushing dynamic that makes them reach layers 4 and 5 much sooner than the drug simulations.

We gathered the layer-specific Growth Index by calculating the AUCs of the median of the 10 replicates. We saw that an administration of drugs on the boundaries leads to varying growth indices among the tumour sphere layers (Fig. 8b). Cells in the central tumour regions do not show any signs of growth inhibition as they have slightly positive growth indices whereas cells on the tumour boundaries show high growth inhibition values with negative growth indices. In summary, the results of these experiments respond to two different dynamics: first, by uptaking drug from the environment, cells in the outer layers have a shielding or protective effect on cells in the inner layers; second, the death of the cells exposed to drugs in the outer layers free up space for the proliferation of cells in the inner layers, even in the innermost layer where space is the scarcest.

## Discussion

PhysiBoSS 2.0 is a redesign and reimplementation of PhysiBoSS to be a reusable, extensible and updatable add-on for the PhysiCell framework. The new add-on design of PhysiBoSS 2.0 allows us to extend its functionalities, reuse in a more efficient way compartmentalised functions and replicate simulations^32^. Our tool follows a modular design that decouples PhysiCell and MaBoSS codes, minimising the dependencies between these tools and ensuring long-term maintainability and is, therefore, more respectful of the single responsibility principle and the façade pattern of software design^40^. This modular design ensures long-term sustainability as either tool (PhysiCell and MaBoSS) can now be updated independently. Furthermore, we also include several sample projects with different degrees of complexity that can be used as templates to develop new models. In addition, we also provide the PCTK python package to ease the task of handling and processing simulation outputs and to easily create summary plots and 3D rendered figures.

We show added functionalities that enable PhysiBoSS 2.0 to study disease mechanisms that cause malfunctions in personalised models while being flexible to handle many different models and consistent with the results of the former PhysiBoSS. As a showcase example, we have here presented a drug screening study that uncovered synergies between drugs and heterogeneities in the cell population and that expands our work done on prostate Boolean models^31^. We have presented drug screening studies that could mimic, albeit simplistically, in vitro spheroid experiments and have validated some of them using real-time cell survival assay data. Implicitly, this work also showcases the flexibility of PhysiBoSS 2.0 to run with any user-provided Boolean model in MaBoSS or SBML-qual format, as opposed to the original PhysiBoSS.

Additionally, PhysiBoSS 2.0 allows the simulation of two potential causes of tumoural heterogeneity: the uneven drug penetration in the tumour caused by the microenvironment and the different genetic backgrounds of the tumour cells. The study of this kind of heterogeneities is needed to have real-size digital twins^20^ that allow for the understanding of the mechanisms behind drug treatment evasion. Current approaches to predict drug synergies do not consider the complexity and heterogeneity typically found in a tumour, even though it is known that these greatly affect the drug effectiveness^41–43^.

As drug administrations are never completely homogeneous but rather occur from the tumour surroundings or from specific points in the tumour environment, such as blood vessels, we will continue studying in upcoming works the growth heterogeneities and the apparent shielding effect that cells from the outer layers can have on other cells from the inner layers to deliver a realistic digital twin of drug treatment. With these added capabilities, PhysiBoSS 2.0 takes important steps towards having truly personalised digital twins that could be used for realistic clinical trials of drug treatments and effective drug synergies. We are currently working on expanding this tool to include the extracellular matrix and its interactions with the cells^28^, blood vessels and their vascularisation and complex three-dimensional architectures taken from spatial omics of patients. Additionally, we are also considering expanding this work with the recent inclusion of PK/PD models in PhysiCell^44^.

Altogether, the new design and implementation allows PhysiBoSS 2.0 to be model-agnostic and easily customisable by users and provides a simple framework of custom modules and custom settings to use with any Boolean model of interest in MaBoSS or SBML-qual format. We have made efforts to provide full accessibility to PhysiBoSS 2.0 code as well as to several accompanying interoperable tools that make the full software bundle reusable and expansible at https://github.com/PhysiBoSS/.

## Methods

### Boolean models

In this work, we have used different Boolean models. The first model is focused on the effect of TNF presence on cell fate decisions, was used previously in PhysiBoSS 1.0 and is an extension of a published Boolean model of cellular fates^3^. It has 31 total nodes, 25 internal nodes, 3 input nodes (*TNF*, Tumour Necrosis Factor Receptor; *FADD*, FAS-associated death domain protein; *FASLG*, Tumour Necrosis Factor Ligand Superfamily Member 6) and 3 output nodes (*Survival*, *Non-Apoptotic Cell Death* (NonACD), *Apoptosis*). The second model is a general model of prostate cancer^45^ that was used to identify potential drug targets in prostate cancer that were personalised to patients and cell lines using different *omic* datasets^31^. The model includes a total of 133 nodes of which 9 correspond to input nodes and 6 to output nodes. The personalised version of the model includes six prostate cancer cell lines (LNCaP^46^, 22Rv1^47^, BPH1^48^, DU145^49^, PC3^50^ and VCaP^51^), the results here presented were obtained using LNCaP. All the models are available in the dedicated repository: https://github.com/PhysiBoSS/Boolean-models.

### Personalisation of Boolean models

Boolean models can be used to simulate the effect of therapeutic interventions and predict the expected efficacy of candidate drugs on different genetic and environmental backgrounds by using our PROFILE_v2 methodology^31^. Herein, the prostate Boolean model was tailored to different datasets using PROFILE_v2 methodology to obtain personalised models that capture the particularities of a set of patients^14^ and of cell lines^15^. Proteomics, transcriptomics, mutations and copy number alteration (CNA) data can be used to modify different variables of the MaBoSS framework, such as node activity status, transition rates and initial conditions. The resulting ensemble of models is a set of personalised variants of the original model that can show great phenotypic differences. Different strategies (use of a given data type to modify a given MaBoSS variable) can be tested to find the combination that better correlates to a given clinical or otherwise descriptive data. In the present case, prostate-cell-line-specific models were built using mutations, CNA and RNA expression data. More details on the PROFILE methodology can be found in its own works^14,31^ and its dedicated GitHub repository: https://github.com/PhysiBoSS/PROFILE_v2. Note that apart from using omics data to personalise the Boolean model, PhysiBoSS 2.0 can also include cell-line-specific phenotypic data, for instance doubling times, to tailor the simulations to a desired behaviour (Supplementary Section 1.5).

### Simulating drug inhibition synergies in the prostate model

In PhysiBoSS 2.0, diffusing drugs in the microenvironment target and inhibit specific input nodes in the Boolean model within the individual cell agents. As modellers, the goal is to identify drugs that target candidate nodes and specific treatment strategies that together might be relevant in producing desired therapeutic outcomes such as increased apoptosis or reduced proliferation. To illustrate how PhysiBoSS 2.0 can be used to conduct in silico drug screening experiments, we select a list of target nodes from^31^ that have yielded exciting results using prostate Boolean models. Suitable drugs are then identified using the DrugBank database^52^ and matched with their availability on the Genomics of Drug Sensitivity in Cancer (GDSC) database^53^. The drug-target pairs of interest for LNCaP can be found in Table 2.

We then integrate the estimated dose–response curves (see section “Estimation of drug-response for single and double drug studies”) into PhysiBoSS 2.0 to model the effect that the local concentration of a drug in the nearby surroundings of a cell has on the intracellular signalling model. Specifically, a cell agent is informed of the drug concentration for each simulated substrate at its nearest voxel. Then, the corresponding target node is inhibited with a probability calculated based on the dose-response curve for the selected drugs (Table 2). The probability of inhibiting a node in a given cell is equal to 1 − *f*([*X*]_*i*_) where [*X*]_*i*_ is the concentration of the drug in voxel *i* and *f* is the normalised dose-response fitted function.

We simulated the inhibition of six nodes of interest on the LNCaP model using PhysiBoSS 2.0. MaBoSS, integrated into PhysiBoSS 2.0, can perform simulations changing the proportion of activated and inhibited status of a given node. For instance, out of 5000 trajectories of the Gillespie algorithm^54^, MaBoSS can simulate 70% of them with an activated AKT and 30% with an inhibited AKT node. Then, the phenotypes’ probabilities for the 5000 trajectories are averaged and these are considered to be representative of a model with a drug that reduces the activity of AKT by 30%.

Using PhysiBoSS 2.0, we simulated the growth of a three-dimensional spheroid with 1138 initial cells of LNCaP prostate cell line for 7 days under different conditions that include the spheroid being treated with six different drugs individually supplied (see Table 2 as well as the spheroid’s growth without any drug, considered as the wild type (WT) conditions. Furthermore, we tested five different drug concentrations for each drug corresponding to the IC10, IC30, IC50, IC70 and IC90. In all the in silico experiments, drugs diffused uniformly from the simulation boundaries. Finally, to validate the results, we took a snapshot of the current state of the simulation every 240 min to create a growth curve for each condition and replicate (Supplementary Fig. 8). The median area under the curve (AUC) for 10 replicates was used to compare growth behaviours upon drug administration. Based on these AUC we defined a Growth Index (*GI*) as follows:

1 $$GI={\log }_{2}\frac{AUC(withdrug)}{AUC(withoutdrug)}$$

A *GI* below zero indicates a reduction in growth upon drug treatment with respect to the untreated condition while a value over zero indicates an increase in growth.

### Estimation of drug-response for single and double drug studies

The effect of a drug on the cell depends on its concentration and provides insight into multiple drug characteristics such as potency or efficacy^55^. Usually, this relation exhibited is non-linear and thus, sigmoidal-shaped functions such as the Hill equation are used to model the dose-response. Herein, we have used standard pharmacodynamics methods to model the effect of various concentrations of drugs on cell line growth. For this purpose, we used GDSC dose-response data (see Table 2) to fit the raw cell viability experiments using a multi-level fixed effect model^38^ implemented in the *gdscIC50 R* package (https://github.com/CancerRxGene/gdscIC50). As a result, we obtained a specific sigmoidal dose-response curve for each drug and cell line pair (see Supplementary Fig. 7).

Drug synergies were studied using the Bliss independence model^56^, which is based on the idea that the two studied compounds are acting independently from each other, meaning that they are non-interacting^57^. Based on the effects of every single drug, a reference model was calculated as follows:

2 $${\hat{E}}_{XY}={E}_{X}+{E}_{Y}-{E}_{X}\cdot {E}_{Y}$$

where ${\hat{E}}_{XY}$ is the predicted combined effect of how the two drugs *X* and *Y* act if no synergy or antagonism exist; whereas 0 ≤ *E*_*X*_, *E*_*Y*_ ≤ 1 are the single drug effects of *X* and *Y*, respectively. If the measured combined drug effect observed is higher than the predicted effect ${\hat{E}}_{XY}$, synergy is declared and antagonism is concluded otherwise. This can also be expressed using the Combination Index (CI)^58^ which is calculated as follows:

3 $$CI=\frac{{\hat{E}}_{XY}}{{E}_{XY}}$$

where *E*_*XY*_ is the inhibition resulting from the double drug simulations. A Combination Index (CI) below 1 indicates synergy while a value above 1 indicates antagonism.

### Computational resources

All the simulations done in this paper were performed in the MareNostrum 4 supercomputer, located at the Barcelona Supercomputing Center in Spain. Each node contains two Intel Xeon Platinum 8160, each one with 24 processors running at 2.1 GHz and 33 MB L3 Cache. Memory is organised in two NUMA sockets with a total amount of 96GB per node. Individual simulations were done using all the 48 CPUs of one compute node. Model exploration performed using EMEWS was carried out using 10 nodes allocating three instances per node and assigning 16 CPUs per simulation instance. Results were analysed using custom scripts written in Python (3.9) that used the PCTK module (see “Handling and processing simulation outputs” in the “Results” section). All the software tools and their repositories used in the present work are listed in Supplementary Table 2 and the ones related to PhysiBoSS in Table 3.

**Table 3:** Codes related with PhysiBoSS with their maintainers and repositories.

Name	Repository	Maintainer
PhysiCell	https://github.com/MathCancer/PhysiCell	Indiana University
MaBoSS	https://github.com/sysbio-curie/MaBoSS-env-2.0	Institut Curie
PhysiBoSS 1.0	https://github.com/PhysiBoSS/PhysiBoSSv1	Deprecated
PhysiBoSS 2.0	https://github.com/PhysiBoSS/PhysiBoSS	BSC, Institut Curie
BioFVM	https://github.com/MathCancer/BioFVM	Deprecated
PCTK	https://github.com/PhysiBoSS/pctk	BSC

### Reporting summary

Further information on research design is available in the Nature Research Reporting Summary linked to this article.

### Supplementary information

PhysiBoSS 2.0: a sustainable integration of stochastic Boolean and agent-based modelling frameworks – Supplementary Text

Reporting Summary

## Data Availability

No new experimental data was generated as part of this study. All the models used in this study are available at https://github.com/PhysiBoSS/Boolean-models. The model used to simulate TNF dosage is an extension from the one from ref. ^3^, and the models of prostate cancer come from ref. ^31^. The drug dosage experiments were reported in ref. ^31^.

## Code availability

The codes used in this study are available at https://github.com/PhysiBoSS. The code for PhysiBoSS 2.0 can be found in https://github.com/PhysiBoSS/PhysiBoSS. The personalisation methodology is available at https://github.com/PhysiBoSS/PROFILE_v2. The PhysiCell ToolKit (PCTK) can be found in https://github.com/PhysiBoSS/pctk. The gdscIC50 R package used to fit sigmoid curves on the cell lines response to drugs is available in its own repository: https://github.com/CancerRxGene/gdscIC50. Other codes of reference for this work can be found in Table 3 and Supplementary Table 2.
