# Supplementary material for: PhysiBoSS 2.0: a sustainable integration of stochastic Boolean and agent-based modelling frameworks
Source: NPJ Syst Biol Appl. 2023 Oct 30;9:54. doi: 10.1038/s41540-023-00314-4 (PMC10616087; doi:10.1038/s41540-023-00314-4)
Supplement: Supplementary file 2 — Reporting Summary [file 41540_2023_314_MOESM2_ESM.pdf]

nature portfolio

Corresponding author(s): Alfonso Valencia, Barcelona Supercomputing Center (BSC), alfonso.valencia@bsc.es

Last updated by author(s): Oct 6, 2023

Reporting Summary

Nature Portfolio wishes to improve the reproducibility of the work that we publish. This form provides structure for consistency and transparency in reporting. For further information on Nature Portfolio policies, see our Editorial Policies and the Editorial Policy Checklist.

Statistics

For all statistical analyses, confirm that the following items are present in the figure legend, table legend, main text, or Methods section.

n/a	Confirmed	
☐	☒	The exact sample size (*n*) for each experimental group/condition, given as a discrete number and unit of measurement
☐	☒	A statement on whether measurements were taken from distinct samples or whether the same sample was measured repeatedly
☐	☒	The statistical test(s) used AND whether they are one- or two-sided<br>*Only common tests should be described solely by name; describe more complex techniques in the Methods section.*
☐	☒	A description of all covariates tested
☐	☒	A description of any assumptions or corrections, such as tests of normality and adjustment for multiple comparisons
☐	☒	A full description of the statistical parameters including central tendency (e.g. means) or other basic estimates (e.g. regression coefficient) AND variation (e.g. standard deviation) or associated estimates of uncertainty (e.g. confidence intervals)
☐	☒	For null hypothesis testing, the test statistic (e.g. *F*, *t*, *r*) with confidence intervals, effect sizes, degrees of freedom and *P* value noted<br>*Give P values as exact values whenever suitable.*
☒	☐	For Bayesian analysis, information on the choice of priors and Markov chain Monte Carlo settings
☒	☐	For hierarchical and complex designs, identification of the appropriate level for tests and full reporting of outcomes
☒	☐	Estimates of effect sizes (e.g. Cohen's *d*, Pearson's *r*), indicating how they were calculated

Our web collection on statistics for biologists contains articles on many of the points above.

Software and code

Policy information about availability of computer code

Data collection	As described in the "Data availability" section: No new experimental data was generated as part of this study. All the models used in this study are available at https://github.com/PhysiBoSS/Boolean-models. The model used to simulate TNF dosage is an extension from the one from reference [3] (https://doi.org/10.1371/journal.pcbi.1000702), and the models of prostate cancer come from reference [31] (https://doi.org/10.7554/eLife.72626). The drug dosage experiments were reported in reference [31] (https://doi.org/10.7554/eLife.72626).
Data analysis	As described in the "Code availability" section: The codes used in this study are available at https://github.com/PhysiBoSS. The code for PhysiBoSS 2.0 can be found in https://github.com/PhysiBoSS/PhysiBoSS. The personalisation methodology is available at https://github.com/PhysiBoSS/PROFILE_v2. The PhysiCell ToolKit (PCTK) can be found in https://github.com/PhysiBoSS/pctk. The gdscIC50 R package used to fit sigmoid curves on the cell-lines response to drugs is available in its own repository: https://github.com/CancerRxGene/gdscIC50.

For manuscripts utilizing custom algorithms or software that are central to the research but not yet described in published literature, software must be made available to editors and reviewers. We strongly encourage code deposition in a community repository (e.g. GitHub). See the Nature Portfolio guidelines for submitting code & software for further information.

Data

Policy information about availability of data

All manuscripts must include a data availability statement. This statement should provide the following information, where applicable:

- Accession codes, unique identifiers, or web links for publicly available datasets
- A description of any restrictions on data availability
- For clinical datasets or third party data, please ensure that the statement adheres to our policy

No new experimental data was generated as part of this study. All the models used in this study are available at https://github.com/PhysiBoSS/Boolean-models. The model used to simulate TNF dosage is an extension from the one from reference [3] (https://doi.org/10.1371/journal.pcbi.1000702), and the models of prostate cancer come from reference [31] (https://doi.org/10.7554/eLife.72626). The drug dosage experiments were reported in reference [31] (https://doi.org/10.7554/eLife.72626).

Research involving human participants, their data, or biological material

Policy information about studies with human participants or human data. See also policy information about sex, gender (identity/presentation), and sexual orientation and race, ethnicity and racism.

Reporting on sex and gender	Not applicable, no human participant was involved in the study. This information has not been collected.
Reporting on race, ethnicity, or other socially relevant groupings	Not applicable, no human participant was involved in the study. This information has not been collected.
Population characteristics	Not applicable, no human participant was involved in the study.
Recruitment	Not applicable, no human participant was involved in the study.
Ethics oversight	Not applicable, no human participant was involved in the study.

Note that full information on the approval of the study protocol must also be provided in the manuscript.

Field-specific reporting

Please select the one below that is the best fit for your research. If you are not sure, read the appropriate sections before making your selection.

☒ Life sciences ☐ Behavioural & social sciences ☐ Ecological, evolutionary & environmental sciences

For a reference copy of the document with all sections, see nature.com/documents/nr-reporting-summary-flat.pdf

Life sciences study design

All studies must disclose on these points even when the disclosure is negative.

Sample size	Not applicable, no human participant was involved in the study. We simulates spheroids of cell lines.
Data exclusions	No data were excluded from the simulations.
Replication	We performed 10 replicates of the simulations of drug on cell lines. No statistical test was used to define this replication size. This size was sufficient to overcome the intrinsic stochasticity of the simulation tools.
Randomization	Not relevant to the study as the cell lines have well-defined distinctive biology.
Blinding	Blinding is not relevant to the study as the goal was to simulate cell line growth and its differencial response to drug assays.

Reporting for specific materials, systems and methods

We require information from authors about some types of materials, experimental systems and methods used in many studies. Here, indicate whether each material, system or method listed is relevant to your study. If you are not sure if a list item applies to your research, read the appropriate section before selecting a response.

Materials & experimental systems

n/a	Involved in the study	
☒	☐	Antibodies
☒	☐	Eukaryotic cell lines
☒	☐	Palaeontology and archaeology
☒	☐	Animals and other organisms
☒	☐	Clinical data
☒	☐	Dual use research of concern
☒	☐	Plants

Methods

n/a	Involved in the study	
☒	☐	ChIP-seq
☒	☐	Flow cytometry
☒	☐	MRI-based neuroimaging
